# ﻿*Raphiocarpustaygiangensis* (Gesneriaceae), a new species from central Vietnam

**DOI:** 10.3897/phytokeys.218.96511

**Published:** 2023-01-10

**Authors:** Cuong Huu Nguyen, Khoa Van Phung, Khang Sinh Nguyen, Leonid V. Averyanov, Vuong Ba Truong, Chu Van Tran, Hai Xuan Cao, Quan Ngoc Chu, Hau Bich Thi Vu, Thoa Kim Thi Pham

**Affiliations:** 1 Vietnam National University of Forestry, Xuan Mai, Chuong My, Ha Noi, Vietnam Vietnam National University of Forestry Ha Noi Vietnam; 2 Institute of Ecology and Biological Resources, Vietnam Academy of Science and Technology, 18 Hoang Quoc Viet Road, Nghia Do, Cau Giay, Hanoi, Vietnam Institute of Ecology and Biological Resources, Vietnam Academy of Science and Technology Hanoi Vietnam; 3 Komarov Botanical Institute Russian Academy of Sciences, Prof. Popov str., 2, St. Petersburg, 197376, Russia Komarov Botanical Institute Russian Academy of Sciences St. Petersburg Russia; 4 Institute of Tropical Biology Vietnam Academy of Science and Technology, 85 Tran Quoc Toan, District 3, Ho Chi Minh City, Vietnam Institute of Tropical Biology Vietnam Academy of Science and Technology Ho Chi Minh Vietnam; 5 Ba Vi National Park, Tan Linh, Ba Vi, Hanoi, Vietnam Ba Vi National Park Hanoi Vietnam; 6 Department of agriculture and rural development, Hai Chau District, Danang City, Vietnam Department of agriculture and rural development Danang Vietnam; 7 The University of Danang – University of Science and Technology, 54 Nguyen Luong Bang str., Lien Chieu District, Danang City, Vietnam The University of Danang – University of Science and Technology Danang Vietnam

**Keywords:** endemism, flora of eastern Indochina, new taxon, plant diversity, plant taxonomy

## Abstract

*Raphiocarpustaygiangensis*, a new species of Gesneriaceae family discovered in Tay Giang District, Quang Nam Province, Central Vietnam, is here described and illustrated. The new species is diagnosed by the combination of its stem up to 2 m long, sericeous hairs on young stem, leaf petiole and adaxial mid-vein, sparsely and minutely serrate leaf margin, axillary inflorescence spreading along stem, sparsely long gland-tipped hairs on peduncle, pedicel, calyx, outside corolla and pistil, calyx 5-disparted from base, purplish white flower with purple stripes inside corolla tube, and dish-shaped stigma formed by 2 semi-orbicular lobes horizontally expanding. Distinct features of the new species and its morphologically closest congener, *Rhaphiocarpusaxillaris*, are compared and discussed. The conservation status of the described species is estimated as Vulnerable (VU D2) according to the IUCN Red List Criteria.

## ﻿Introduction

The genus *Raphiocarpus*Chun (1946) from Gesneriaceae family includes about 16 species, distributed from southwest China (Weber et al. 1998, 2020; Li and Wang 2005; Wei et al. 2010; Zhang et al. 2010; Chen et al. 2015; Wei 2018; Wei et al. 2022) to central Vietnam (Pham 2000; Phuong 2005; Phuong and Xuyen 2010; Phuong et al. 2012; Luu et al. 2018; Vu 2018; Middleton et al. 2021; Hassler 2022; Powo 2022; Xin et al. 2022). There are presently ten species of *Raphiocarpus* found and described in Vietnam, namely *R.annamensis* (Pellegr.) B.L.Burtt (Weber et al. 1998), *R.asper* (Drake) B.L.Burtt (Weber et al. 1998), *R.axillaris* D.J.Middleton (Middleton et al. 2021), *R.begoniifolius* (Levl.) Burtt (Weber et al. 1998), *R.clemensiae* (Pellegr.) B.L.Burtt (Weber et al. 1998), *R.evrardii* (Pellegr.) B.L.Burtt (Weber et al. 1998), *R.macrosiphon* (Hance) Burtt (Weber et al. 1998), *R.petelotii* (Pellegr.) B.L.Burtt (Weber et al. 1998), *R.tamdaoensis* Phuong Xuyen & Y.G.Wei (Phuong et al. 2012) , and *R.sinovietnamicus* Z.B.Xin, L.X.Yuan & T.V.Do (Xin et al. 2022).

During the botanical fieldwork in Quang Nam Province, Tay Giang District, A Xan Village in central Vietnam in April 2022, we collected several samples of Gesneriaceae. These plants have subshrub habit, opposite leaves spreading along stem, axillary 1–3-flowered cyme, 5-lobed calyx dissected from the base, 4 fertile stamens arranged in two pairs, and 2-lobed stigma, which allows us to identify them as a representatives of the genus *Raphiocarpus.* After consulting the relevant literatures (Pellegrin 1930; Chun 1946; Wang et al. 1998; Weber et al. 1998; Ho 2000; Wei et al. 2010; Zang et al. 2010; Phuong et al. 2012; Chen et al. 2015; Luu et al. 2018; Vu 2018; Wei 2018; Middleton et al. 2021; Wei et al. 2022; Xin et al. 2022) and examining *Raphiocarpus* specimens housed in such herbaria as E, K, P, LE, PE, IBK, KUN, and VNMN, we assume our plants as a new species, well segregated from all known species of the genus by its morphological characters. This discovered new species is described and illustrated below.

## ﻿Materials and methods

All collected and studied herbarium specimens of the newly discovered species are presently stored in the herbaria of Vietnam (HN, VNF) and Russia (LE). Color photos of plants were taken in natural habitats. Morphological observations and measurements were made on living plants, dried specimens, and on alcohol preserved materials. Morphological characters were described using the terminology proposed by Wang et al. (1998)Harris and Harris (2001).

## ﻿Taxonomic treatment

### ﻿ Raphiocarpus taygiangensis

Taxon classificationPlantaeLamialesGesneriaceae

C.H.Nguyen, K.S.Nguyen & Aver. sp. nov.

484695B6-0C88-5176-8567-2ACE57CF5F55

urn:lsid:ipni.org:names:77311676-1

Figs 1 , 2 , 3

#### Diagnosis.

The new species differs from closest *R.axillaris* in serrulate leaves, purple spots and glandular hairs on abaxial surface of corolla, purple longitudinal stripes on median lobe of lower lip, and pubescent filaments stamens and pistil (Table 1).

**Table 1.** Most significant morphological discriminative characters of *Raphiocarpustaygiangensis* and *R.axillaris*.

Characters	*R.taygiangensis*	*R.axillaris*
Stems	to 3 m long, ascending to 1 m tall	to 0.7 cm long and tall
Leaf margin	serrulate	Entire
Corolla	white with purple tint to purple, inside with purple lines; glandular-hairy inside	white to pale pink; glabrous inside
Stamens	4–5; filaments puberulent	4; filaments, glabrous
Staminodes	hooked	clavate
Pistil	puberulent	glabrous

#### Type.

Vietnam. Quang Nam Province, Tay Giang District, A Xan Village, primary evergreen broad-leaved forest, around point 15°48'57"N, 107°19'47"E, elevation 1270 m, 20 April 2022, *C.H. Nguyen*, *K.S. Nguyen*, *H.X. Cao*, *CKH 2022042068* (holotype VNF; isotypes HN, LE).

#### Description.

Perennial herb with stem to ca. 3 m long ascending to ca.1 m tall. Stem branching, velutinous when young, glabrescent with age. Leaves opposite, equal to unequal in size; petioles 1.5–3.5 cm long, densely hirsute with appressed hairs; leaf blade symmetrically elliptic, 8–16 × 3.4–6.2 cm, 2.3–2.6 times as long as wide, base cuneate, apex acute to acuminate, hirsute with appressed hairs on both sides, more densely on veins, serrulate along the margin, eucamptodromous venation with 8–14 pairs of secondary veins, tertiary venation ramified. Inflorescences arising in axils of lower leaves and in leaf scar axils, 1- or 2-flowered, up to 3 inflorescences in an individual axil, 5–6 cm long (including flower); all axes with 0,5–1mm long glandular hairs; peduncle 10–18 mm long; bracts narrowly elliptic, 4–5 × 0.8–1 mm long, greenish, with 0,5–1mm long glandular hairs; flowers spreading almost horizontally to slightly pendulous; pedicels 10–12 mm long. Calyx of 5 lobes free from the base, lobes 6–7 × 1.3–1.5 mm long, with long glandular hairs outside, glabrous inside. Corolla infundibular, outside white with purple tint to purple, inside white with purple on adaxial lip and purple longitudinal lines on median lobe of abaxial lip, 4.2–4.8 cm long, sparsely covered with long glandular hairs outside, inside with glandular short hairs at apex of adaxial lip, with two to three prominent ridges ventrally at the base of throat, limb distinctly two-lipped; tube 3.5–3.9 cm long, 8–10 mm wide, swelling at middle and narrowing at base, the distal part broadening towards throat; adaxial lip 2-lobed, lobes subequal, half round, 5–6 mm long, 5–6 × 7–9 mm wide, sinus 4–5.5 mm deep; adaxial lip 3(4)-lobed, lobes unequal, 14–16.5 mm long, lateral lobes 6–8 × 7–9 mm wide, middle lobe 7–9.2 × 4.8–6.1 mm wide. Stamens 4(5), in 2 pairs, each pair adnate at the anthers, filaments filiform, distally shortly glandular puberulent, geniculate near the middle; adaxial pair adnate to 14–16 mm above the corolla base, 9–11 mm long, 1 mm in diameter, anthers 0.8–1 × 1.1–1.3 mm long; abaxial pair, adnate at c.14 mm above the corolla base, 6–8 mm long, 0.8 mm in diameter; staminode 1, slightly hooked, 3–4 mm long. Disc circular, light lemon yellow, 1–1.2 mm high, margin repand, glabrous. Pistil 2–2.2 cm long, puberulent throughout; ovary 12–13 mm long; style 6–7 mm long; stigma c. 2 mm, 2-lobed. Capsule green when young, bent at the base, narrowly fisiform, pubescent, dehiscing adaxially, straight, not twisted.

**Figure 1.**
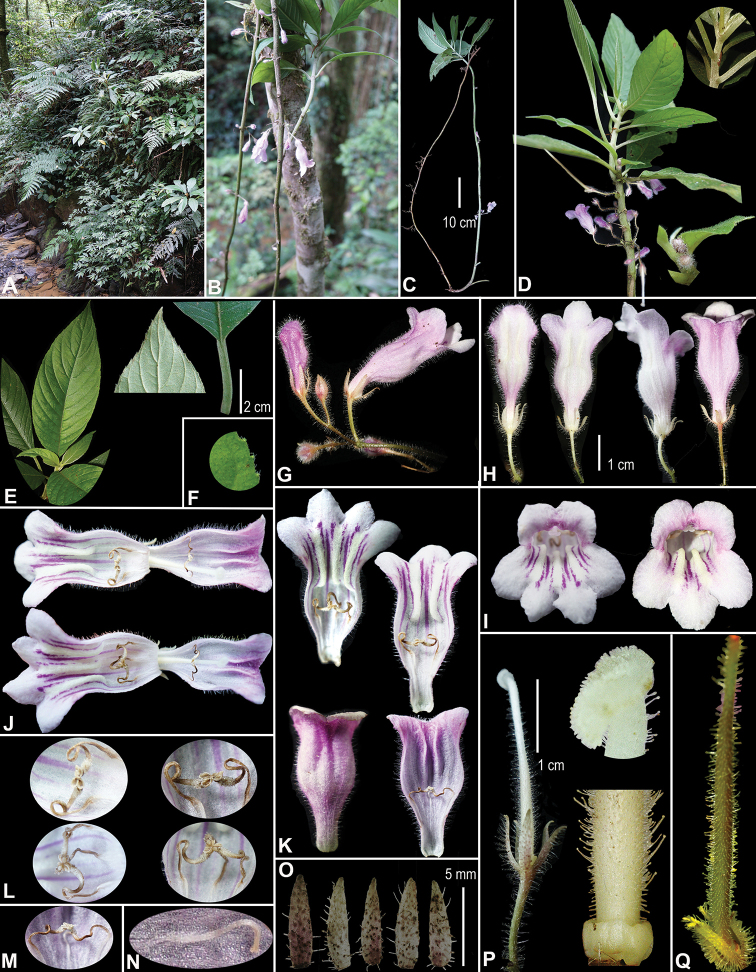
*Raphiocarpustaygiangensis* C.H.Nguyen, K.S.Nguyen & Aver., sp. nov. **A** natural habitat **B–D** flowering plant in natural habitat **E** leaves **F** leaf margin **G** inflorescences **H** flowers, views from different sides **I** flower, frontal views **J** sagittal section of corolla showing inner surface with stamens and staminodes **K** flower tube inside and outside **L, M** stamens **N** staminode **O** calyx lobes **P** pistil, base of pistil and its apex with stigma **Q** young fruit. Photos by Cuong Huu Nguyen and Khang Sinh Nguyen, correction and design by Cuong Huu Nguyen.

#### Distribution and habitat.

The new species is only known from A Xan Commune, Tay Giang District, Quang Nam Province in central Vietnam. *Raphiocarpustaygiangensis* usually grows in moist shady places near waterfalls, along streams and occasionally on the sandstone slopes covered by evergreen broad-leaved forests at elevations of 1200–1300 m a.s.l. As common plants in habitats of the new species have been recorded *Aeschynanthusbracteatus* Wall. ex A.DC., *Angiopterisevecta* (G. Forst.) Hoffm., *Aspleniumunilaterale* Lam., *Begonia* spp., *Crepidomanesauriculatum* (Blume) K. Iwats., *Hedyotis* sp., *Impatiensclavigera* Hook. f., *Leptochilus* sp., *Molineriacapitulata* (Lour.) Herb., *Mycetia* sp., *Phymatosoruslucidus* (Roxb.) Pic. Serm., *Rhaphidophoradecursiva* (Roxb.) Schott, *Rhynchotechumellipticum* (Wall. ex D. Dietr.) A. DC., and *Symplocosbanaensis* Guillaumin.

**Figure 2.**
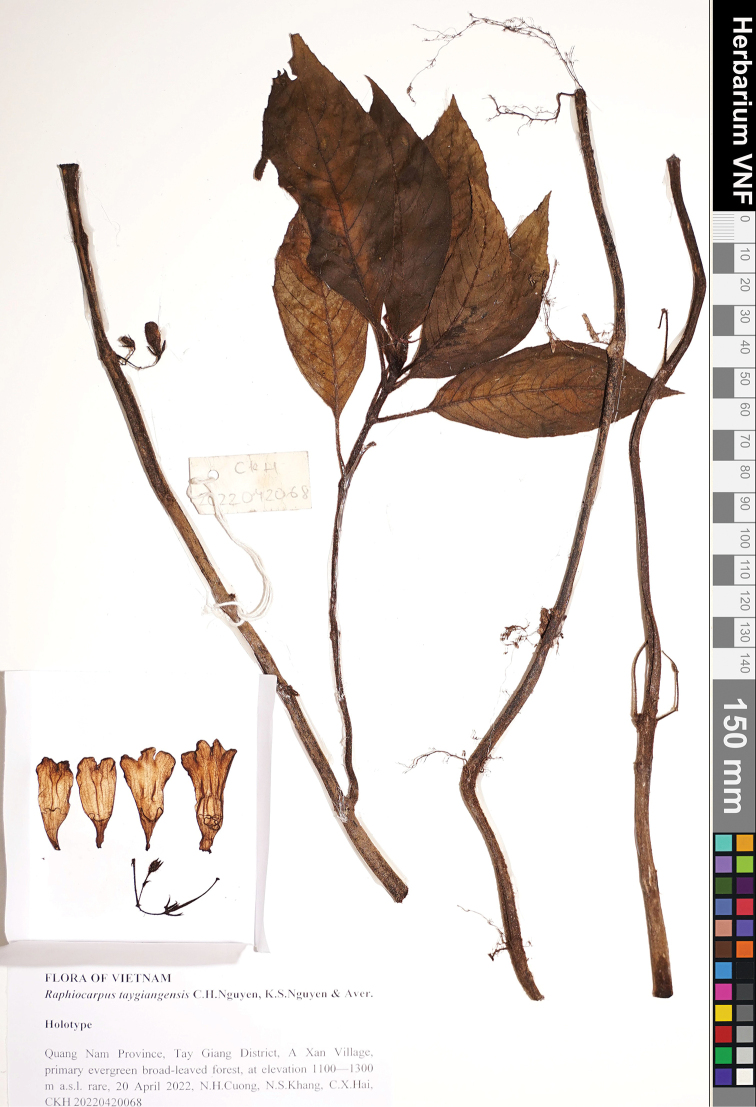
*Raphiocarpustaygiangensis* C.H.Nguyen, K.S.Nguyen & Aver., sp. nov. holotype specimen, Cuong et al., CKH 2022042068 (VNF).

#### Phenology.

Flowers from April to May, fruiting from May to June.

#### Etymology.

The species epithet refers to the name of the district of the type location (Tay Giang District in Quang Nam Province).

#### IUCN conservation status.

The special field studies around the type location revealed no other populations outside the occupancy area which was estimated to be about 5 km^2^. The type location consists of approximately 500 mature individuals growing in moist shady places near waterfall and along the stream. The population territory currently does not belong to any protected area, and its future protection is needed. The area has been relatively undisturbed to date due to its significance to the surrounding village, although local people continue to forage in it for firewood. Considering the small population size and fragile habitat, we propose that the new species should be preliminarily assessed as Vulnerable (VU D2) according to criteria IUCN (2019).

**Figure 3.**
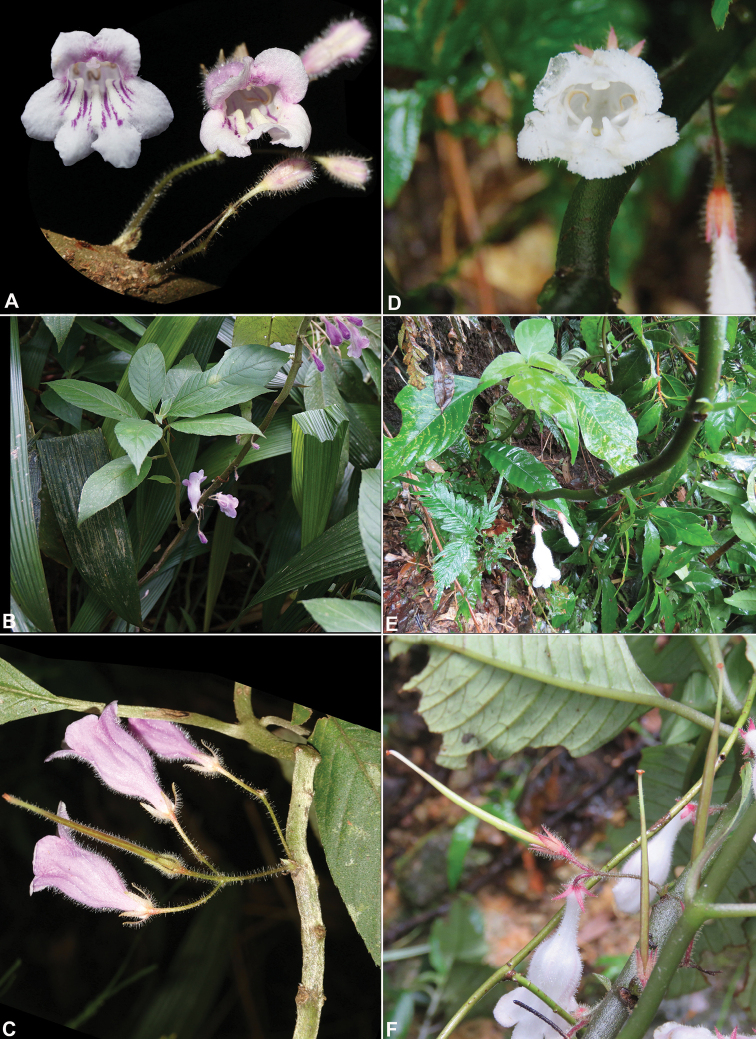
*Raphiocarpustaygiangensis* C.H.Nguyen, K.S.Nguyen & Aver., sp. nov. **A** flower front view **B** habitat **C** lateral view of flower and fruit; *Raphiocarpusaxillaris* D.J.Middleton, sp. nov. **D** flower front view **E** habitat **F** lateral view of flower and fruit. Photos by Ly Van Nguyen (**D–F**), Cuong Huu Nguyen and Khang Sinh Nguyen, correction and design by Cuong Huu Nguyen.

#### Note.

A comparison of the most significant morphological characters of *Raphiocarpustaygiangensis* and its closest congeners, *R.axillaris*. The new species can be easily distinguished from *R.axillaris* in having basally prostrate and distally ascending stem; sericeous hairs on young stem, leaf petiole and adaxial mid-vein; sparsely serrulate leaf margin; axillary inflorescence; sparse glandular hairs on the peduncle, pedicel, calyx, pistil, and abaxial surface of corolla; calyx 5-lobed, dissected from the base; white or light purplish flowers with purple stripes inside corolla tube; twining filaments; and dish-shaped stigma formed by 2 semi-orbicular lobes spreading horizontally. The comparison of the key morphological characters of R.taygiangensis, and *R.axillaris* is presented in Table 1.

## Supplementary Material

XML Treatment for Raphiocarpus taygiangensis
